# Third-Generation Sequencing and Analysis of Four Complete Pig Liver Esterase Gene Sequences in Clones Identified by Screening BAC Library

**DOI:** 10.1371/journal.pone.0163295

**Published:** 2016-10-03

**Authors:** Qiongqiong Zhou, Wenjuan Sun, Xiyan Liu, Xiliang Wang, Yuncai Xiao, Dingren Bi, Jingdong Yin, Deshi Shi

**Affiliations:** 1 State Key Laboratory of Agricultural Microbiology, College of Veterinary Medicine, Huazhong Agricultural University, Wuhan, Hubei, China; 2 Key Laboratory of Development of Veterinary Diagnostic Products of Ministry of Agricultural, College of Veterinary Medicine, Huazhong Agricultural University, Wuhan, Hubei, China; 3 State Key Laboratory of Animal Nutrition, College of Animal Science and Technology, China Agriculture University, Beijing, China; Cornell University, UNITED STATES

## Abstract

**Aim:**

Pig liver carboxylesterase (PLE) gene sequences in GenBank are incomplete, which has led to difficulties in studying the genetic structure and regulation mechanisms of gene expression of PLE family genes. The aim of this study was to obtain and analysis of complete gene sequences of PLE family by screening from a Rongchang pig BAC library and third-generation PacBio gene sequencing.

**Methods:**

After a number of existing incomplete PLE isoform gene sequences were analysed, primers were designed based on conserved regions in PLE exons, and the whole pig genome used as a template for Polymerase chain reaction (PCR) amplification. Specific primers were then selected based on the PCR amplification results. A three-step PCR screening method was used to identify PLE-positive clones by screening a Rongchang pig BAC library and PacBio third-generation sequencing was performed. BLAST comparisons and other bioinformatics methods were applied for sequence analysis.

**Results:**

Five PLE-positive BAC clones, designated BAC-10, BAC-70, BAC-75, BAC-119 and BAC-206, were identified. Sequence analysis yielded the complete sequences of four PLE genes, PLE1, PLE-B9, PLE-C4, and PLE-G2. Complete PLE gene sequences were defined as those containing regulatory sequences, exons, and introns. It was found that, not only did the PLE exon sequences of the four genes show a high degree of homology, but also that the intron sequences were highly similar. Additionally, the regulatory region of the genes contained two 720bps reverse complement sequences that may have an important function in the regulation of PLE gene expression.

**Significance:**

This is the first report to confirm the complete sequences of four PLE genes. In addition, the study demonstrates that each PLE isoform is encoded by a single gene and that the various genes exhibit a high degree of sequence homology, suggesting that the PLE family evolved from a single ancestral gene. Obtaining the complete sequences of these PLE genes provides the necessary foundation for investigation of the genetic structure, function, and regulatory mechanisms of the PLE gene family.

## 1 Introduction

Pig liver carboxylesterase, also known as pig liver esterase (PLE), consists of a family of enzymes composed of various isozymes. PLE is indispensable in the field of organic synthesis owing to its unique asymmetric hydrolytic activity, and nearly a century of research on PLE has centred on its applications in this field [1,2]. PLE can hydrolyse butyrylcholine, aromatic amines, and other exogenous and endogenous esters and amides; therefore, there is a reason to believe that PLE serves an important function in ester metabolism and other porcine physiological processes [3,4], such as drug metabolism and detoxification, and regulation of inflammation levels.

PLE is the most complex family of carboxylesterases in mammals, consisting of many members with similar physical and chemical properties. Hence, it is very difficult to isolate and purify isozymes containing a single component using physical chemistry methods. Extraction of natural PLE from pig liver had already been attempted in the 1960s, but the PLE extracted from pig liver was found to comprise a mixture of multiple isozymes, each with different hydrolytic characteristics [5,6,7]. For these reasons, the investigation and application of PLE was severely hindered. Rapid advances in molecular biology techniques made it possible to obtain single-component PLE samples. Seven isozymes, PLE1-PLE6 and APLE, have been cloned and expressed, and their hydrolysis activity were also explored [8,9,10]. The porcine intestinal carboxylesterase (PICE) which have a high homology with PLE was also cloned、expressed and purified [11,12]. The first cDNA sequence of PLE isoenzymes identified and expressed was γ-PLE [9,13]. Moreover, sequence alignment of these cDNA and amino acid sequences of PLE has revealed an N-terminal signal peptide sequence of 18 amino acids and a C-terminal endoplasmic reticulum retention signal of His-Ala-Glu-Leu. In addition, using the crystal structure of human carboxylesterase as a reference, homology modelling has provided preliminary understanding of the spatial structure of PLE [14].

In our previous study, more than 54 isoenzymes of PLE cDNA were obtained from Tongcheng pig and Large white pig, and from each individual of pig, about 8–15 isoenzymes were found, and the main isoenzymes were also expressed and their hydrolysis activity also were examined, and it turn out that each enzyme has different activity (data wait for publication). Taking trimmer is required for these enzyme to be active [14], the number of different trimmers would be huge in an individual of pig, which confirmed the pig carboxylesterase family is the most complex family. So, we are attracted by the question how the PLE family is organized in genome, and how the gene family is regulated. Although PLE has been widely investigated, gaps in PLE gene sequencing data and knowledge of genetic regulatory mechanisms remain. PLE gene sequences from authoritative databases are either incomplete or not in order, making them an inadequate basis for understanding the genomic structure and gene regulation mechanism of PLE family. In this study, we performed sequence alignment of existing PLE gene and cDNA sequences, revealing that different PLE subtypes not only have a high degree of sequence similarity in their exons, but also a high degree of homology of intron sequences. So, Fragments obtained by conventional PCR, and second-generation gene sequencing methods cannot adequately predict to splice the complete PLE gene sequences.

In this study, we used a previously constructed Rongchang pig BAC genomic library [15], and primers designed from conserved regions of PLE exons to screen for positive BAC clones. Next, third-generation PacBio single-molecule sequencing technology (average read length, 10kb) was employed to identify PLE genes and accurate splice in positive BAC clones (average length, 110kb). Then, BLAST comparisons and other bioinformatics methods were applied for sequence analysis.

## 2 Results

### 2.1 Primer verification

**Fig 1:**
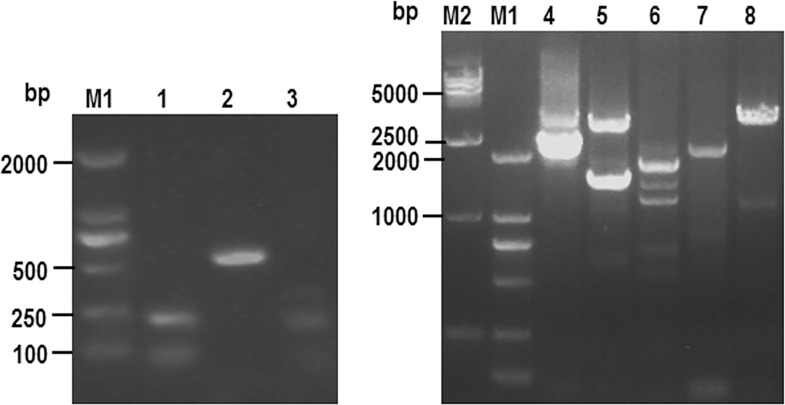
Screening primers for fishing positive clones in BAC library. 1–8: Products of PCR of pig genome by1-8 primers. M1: DL 2000 DNA ladder maker M2: DL 15000 DNA ladder maker.

PCR amplification to validate primer sets was performed using pig genomic DNA as a template and amplification products were characterised by 0.8% agarose gel electrophoresis (Fig 1). Primers pair 1 and 2 both amplified bands of the expected sizes (208 bps and 534 bps, respectively), demonstrating that these primer pairs are useful for screening for PLE-positive clones. Primer pairs 4, 5, and 6 each amplified two bands of approximately the expected sizes, indicating that the sizes of introns 4, 5, and 6 differed among the PLE clones. Hence, primer pairs 4, 5, and 6 can be used in PCR to detect multiple positive plasmids, and if the resulting amplicons are of different sizes, this may indicate that the positive clones differ from one another. Primer pair 3 did not amplify a band of the expected size. Primer pairs 7 and 8 only amplified single dim bands, indicating that these primer pairs cannot be adopted for use in differentiating between positive clones; therefore, they were not used in further analyses in this study. Ultimately, primer pairs 1, 2, 4, 5, and 6 were selected for use in screening for PLE-positive clones in the BAC library.

### 2.2 BAC library screening of PLE-positive clones

**Fig 2:**
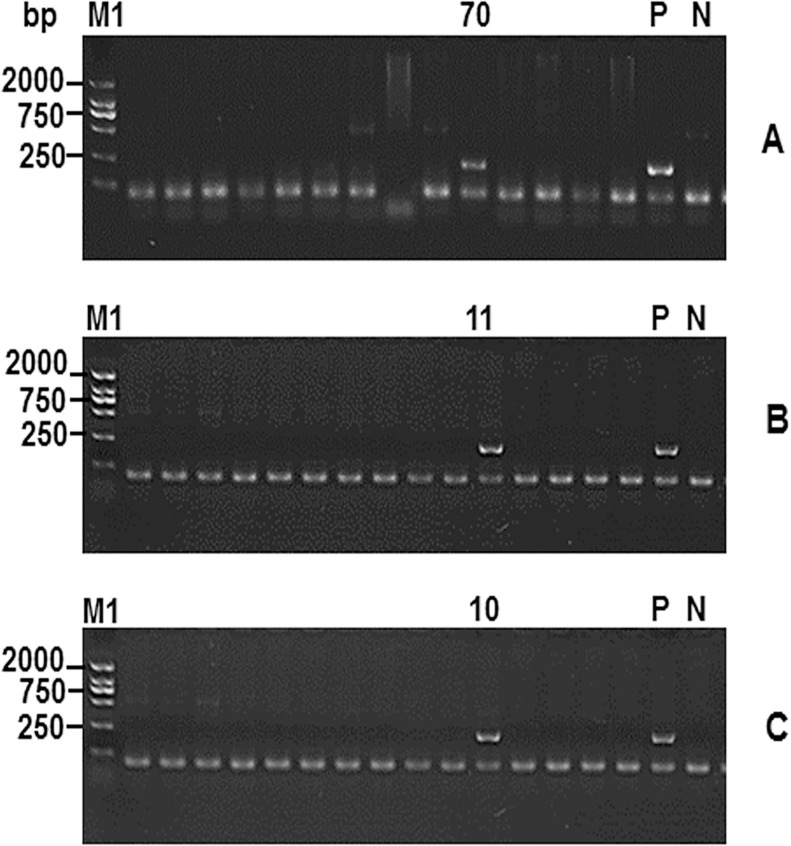
The positive clone BAC-10 was identified using the three-step PCR screening system. Note: (A)The first step was to identify the 70^th^ plate pools had a positive clone. (B)The second step was to screen the 70^th^ plate and the 11^th^ columm had a positive clone. (C) The third step was to screen the the 11^th^ colummof the 70^th^ plate and BAC-10 was identified as a positive clone. (P) was the positive control containing pig genomic DNA. (N) was the negative control containing ddH_2_O. M1: DL 2000 DNA ladder maker.

A three-step PCR screening method was employed, and primer pairs 1, 2, 4, 5, and 6 were used to identify five positive BAC clones from the library, designated BAC-10, BAC-70, BAC-75, BAC-119, and BAC-206. Fig 2 shows the method by which primer pair 1 was used to identify the BAC-10 positive clone, which is representative of other BAC positive clones screening method.

### 2.3 Plasmid isolation and identification

**Fig 3:**
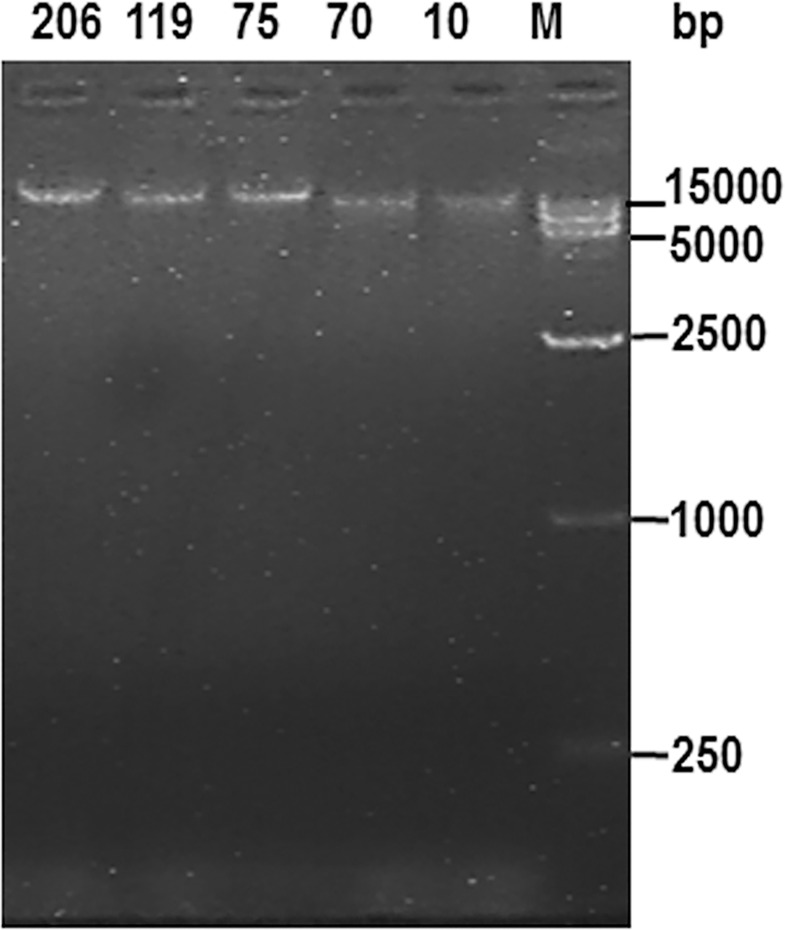
The plasmids of five BAC positive clones were identified by agarose gel electrophoresis. M: DL 15000 DNA ladder maker.

Positive clones (BAC-10, BAC-70, BAC-75, BAC-119, and BAC-206) were transferred to LB liquid culture containing 12.5μg/mL chloramphenicol and BAC DNA isolated according to the instructions provided in the BAC/PAC DNA Isolation Kit. DNA concentration was measured using a *NANODROP* 2000C spectrophotometer (Thermo Scientific). The concentrations of the five clones were 3000-5000ng/μL, the A_260_/A_280_ ratios were 1.91–1.96, and the A_260_/A_230_ ratios were 2.1–2.2. Each isolated clone (5 μl) was subject to electrophoresis on a 0.8% agarose gel (50 V, 4°C, 3 h). Each sample migrated as a complete band without any indication of contamination by impurities or of degradation (Fig 3). Thus the clones met the quality requirements for PacBio third-generation sequencing.

### 2.4 PLE gene sequencing results and bioinformatics analysis

After purification and concentration of the five BAC samples, a library composed of 20kb fragments derived from each sample was constructed. After SMRT sequencing, the original output data was filtered, and high-quality sequences of BAC-10, BAC-70, BAC-75, BAC-119, and BAC-206 were generated, with totals of 41,228,895bps, 1,013,780,514bps, 1,487,229,841bps, 307,626,930bps, and 191,394,912bps, respectively. The average read length of the SMRTcell^TM^ Library was approximately 10kb. The accuracy of the sequencing data was 99.999%, demonstrating its reliability. Bioinformatics was then applied to analyse and assemble the sequences of BAC-10, BAC-70, BAC-75, BAC-119, and BAC-206, and a total of five BAC sequences were obtained, with lengths of 109,591 bps, 127,733 bps, 106,999 bps, 127,294 bps, and 116,854 bps, respectively.

Our previous study obtained 54 PLE subtypes from Tongchong pig and Large White pig (data wait for publication), which were divided into A, B, C, D, E, F, G groups according to the similarity of their amino acid sequence, and among the same group, different isotypes were distinguished by Araba numbers. PLE genes found in this study are named in the same way.

**Fig 4:**
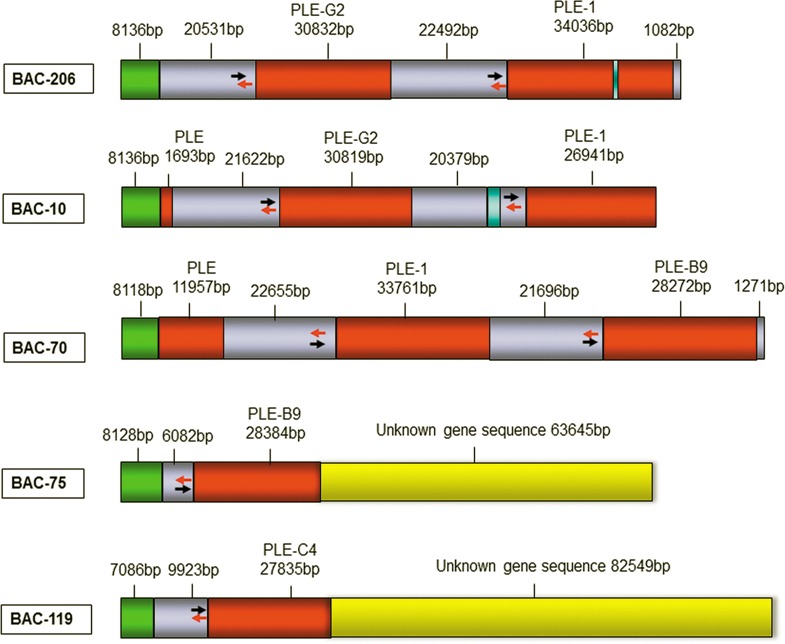
The pattern diagrams of five BAC positive plasmids. Note: Red areas represent the sequences of PLE gene, Gray areas represent the intergenic sequence of PLE gene, Green areas represent the pCC1BAC sequence, Yellow areas represent unknown gene sequence, Orange areas represent partial pig CH242 gene sequence. Blue said missing sequence after comparing, The double arrows (red and black) indicate the reverse complementary sequence of PLE gene regulatory regions.

The sequences of the assembled BAC DNA samples were aligned with that of γ-PLE cDNA (GenBank: X63323) using BLAST. The five BAC sequences were found to contain a total of four complete PLE genes; BAC-10 contained the complete sequence of the PLE-G2 gene, BAC-70 contained two complete PLE genes (PLE-1 and PLE-B9), BAC-75 contained PLE-B9 and an unknown 63kb gene sequence, BAC-119 contained PLE-C4 and an unknown 82kb gene sequence, and BAC-206 contained PLE-G2 and PLE-1. The lengths of the PLE-1, PLE-B9, PLE-C4, and PLE-G2 gene sequences were 33,759bps, 28,384bps (BAC-75) /28,272bps (BAC-70), 27,835bps, and 30,819bps, respectively; these sequences were submitted to GenBank, and their accession numbers are KX577727, KX577728, KX577729, and KX577730, respectively. The average length of the spacer sequences between the PLE genes was approximately 22kb. In addition, sequence alignment revealed that the PLE-B9 genes obtained from BAC-70 and BAC-75 showed 100% amino acid sequence homology (with only one nucleotide difference). Sequence alignment of the regions 6082bps upstream of these genes revealed 99% homology (9 gaps, no mutations), revealing the sequence obtained is reliable. Sequence information for each BAC clone is presented in Fig 4.

Using the γ-PLE cDNA sequence (GenBank: X63323) as a reference, and based on the GT-AG rule of bases at the start and end of introns, the exons, introns, noncoding sequences, and spacer sequences were identified for each PLE gene. The four PLE genes were found to have 14 exons and 13 introns, of which exons 1, 2, 5, 7, 10, 11, 13, and 14 were relatively highly conserved.

**Fig 5:**
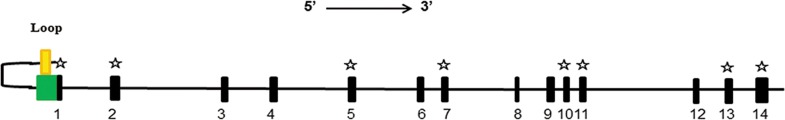
The structure diagram of PLE-1 gene. Note: Black square shows exons, linear shows introns, ☆ shows the conservative exons, arrows indicates gene direction, yellow square shows the reverse complementary sequence which can form a loop rings with the green part.

The regulatory regions of the four PLE genes contained two regions of reverse complement sequence of 720bps in length, taking an example of PLE1, one at nucleotides -5132bp /-4413bp and the other at -802bp /-82bp, separated by 3611bps. These two sequences can be complementary to form Loop ring structure, which is likely to be very significant for the regulation of gene expression. Fig 5 shows the structure of the PLE-1 gene; the structural features of the other three PLE genes are similar.

The regulatory sequences of the different PLE genes exhibited a high degree of homology. Using the regulatory region 6000bps upstream of the PLE-1 gene start codon as a reference, homology with the PLE-G2 upstream sequence was up to 99%, and that with PLE-B9 and PLE-C4 was approximately 96%. In addition, the size and sequence of introns and the sequence of exons of the different PLE genes differed to some extent; however, the sequences of all exon-intron junctions were highly conserved and followed the AG-GT rule. Alignment of the amino acid sequences of the four PLE genes revealed that the homology between the four isoforms and γ-PLE was ≥ 96%, and all had identical signal peptide sequences of 18 amino acids and C-terminal ER retention signals (HAEL). Intron alignment showed that the introns of PLE-1 and PLE-G2 were the most similar; the homologies between all introns were ≥ 97% in these two genes, except for intron 2 because of different sizes (PLE-1 is 5403bps but PLE-G2 is 2195bps). The introns of PLE-B9 and PLE-C4 were relatively similar in size, with homologies of≥ 96%. The introns of the two PLE-B9 genes obtained from BAC-70 and BAC-75 were ≥ 99% homologous. Details of the exon and intron sizes of the four PLE genes are presented in Tables 1 and 2.

**Table 1:** The exons size of four PLE genes (bps) and the amino acids homology with γ-PLE.

GenesID	1	2	3	4	5	6	7	8	9	10	11	12	13	14	amino acids homology
**γ-PLE[9]**	52	208	145	134	154	105	105	39	141	81	148	132	73	181	*
**PLE-1[9]**	52	208	145	134	154	105	105	39	141	81	148	132	73	181	99%
**PLE-B9**	52	208	145	134	154	105	105	39	141	81	148	132	73	181	96%
**PLE-C4**	52	208	145	134	154	105	105	39	141	81	148	132	73	181	96%
**PLE-G2**	52	208	145	134	154	105	105	39	141	81	148	132	73	181	98%

Note: PLE-1 and γ-PLE are reported in the literatures, while PLE-B9, PLE-C4 and PLE-G2 are new ones discovered in this study. But the complete gene of PLE-1, PLE-B9, PLE-C4, PLE-G2 have never been reported.

**Table 2:** The introns size of four PLE genes (bps).

Genes ID	1	2	3	4	5	6	7	8	9	10	11	12	13
**PLE-1**	3021	5403	2621	3739	3511	1153	3995	1068	668	687	4692	1230	281
**PLE-B9(75)**	3009	5440	2586	2265	1481	1806	2205	1069	679	688	4291	883	281
**PLE-B9(70)**	3007	5440	2584	2259	1477	1791	2179	1044	661	685	4282	883	281
**PLE-C4**	2998	5421	2555	2253	1458	1797	2209	1068	663	687	3855	889	281
**PLE-G2**	3013	2195	2628	3753	3522	1155	3998	1079	667	689	4713	1232	281

Note: the PLE-B9 (75) refers to the gene sequences of PLE-B9 from the BAC-75 plasmid and PLE-B9 (70) refers to the gene sequences of PLE-B9 from the BAC-70 plasmid.

**Fig 6:**
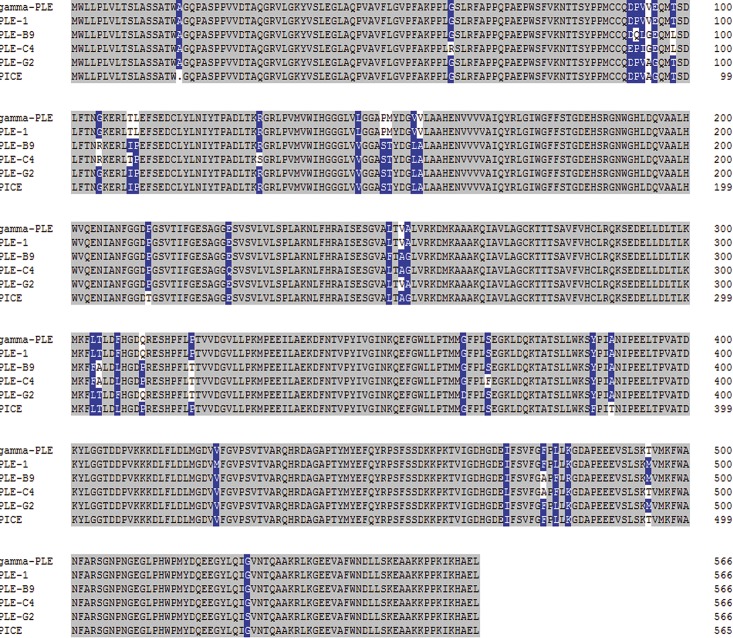
Comparison of amino acids sequences of different isoenzymes. Note: Gray colour represents the same amino acids while the blue and white colour refers to the difference amino acids between, refers to the deleted amino acids.

Meanwhile, a BLASTP comparison between the known γ-PLE isoenzyme with PLE1、PLE-B9、PLE-C4、PLE-G2 isoenzymes and PICE was also performed, which confirmed that the difference amino acids are not randomly distributed in the sequences, but are in several distinct regions with total of 25 AA different [3], PICE was not identical with anyone of them, and PLE1 are identical to γ-PLE, while PLE-B9, PLE-C4, PLE-G2 were not identical to anyone of others (Fig 6).

As the peptide sequence of PLE isoenzymes reported was different in 25 AA positions[3], comparison of the 25 AA positions for the 7 known PLE isoenzyms together with PLE1, PLE-B9, PLE-C4, PLE-G2 isoenzymes was performed. And the results summarized in Table 3 which showed that PLE1 is identical to γ-PLE while PLE-B9, PLE-C4, PLE-G2 are new ones.

**Table 3:** The distribution of different amino acid sequences of PLE isoenzymes.

AA	γ-PLE	PLE2	PLE3	PLE4	PLE5	PLE6	APLE	PLE-1	PLE-B9	PLE-C4	PLE-G2
**73**	D	D	E	D	D	E	E	D	D	E	D
**75**	V	V	I	V	V	I	I	V	I	I	V
**76**	V	V	G	A	A	G	G	V	G	G	A
**77**	E	E	G	G	G	G	G	E	E	E	G
**80**	T	T	L	T	T	L	L	T	L	L	T
**87**	G	G	R	R	R	R	R	G	R	R	G
**92**	T	T	I	I	I	I	I	T	I	T	I
**93**	L	L	P	P	P	P	P	L	P	P	P
**129**	L	L	V	V	V	V	V	L	V	V	V
**133**	P	P	S	S	S	S	S	P	S	S	S
**134**	M	M	T	T	T	T	T	M	T	T	T
**138**	V	V	L	L	L	L	L	V	L	L	L
**139**	V	V	A	A	A	A	A	V	A	A	A
**234**	L	L	F	F	F	F	F	L	F	L	L
**236**	V	V	A	A	A	A	A	V	A	A	V
**237**	A	A	G	G	G	G	G	A	A	A	A
**285**	F	F	P	P	F	F	F	F	F	F	F
**286**	L	L	L	L	F	F	F	L	F	F	L
**287**	T	T	T	T	A	A	A	T	A	A	T
**290**	F	F	L	L	L	L	L	F	L	L	F
**294**	Q	Q	P	P	P	P	P	Q	P	P	Q
**302**	P	P	T	T	T	T	T	P	T	T	T
**459**	F	A	F	A	A	A	F	F	A	A	F
**461**	L	F	L	F	F	F	L	L	F	F	L
**463**	K	R	K	R	R	R	K	K	R	K	K
**Ref.**	[9]	[3]	[3]	[3]	[3]	[3]	[10]				

Note: The position of amino acid was in sequence which signal peptide being deleted. The cDNA sequence of PLE1, PLE-B9, PLE-C4 and PLE-G2 isoenzymes are discovered by our previous study (Data are included in another paper prepared).

Comparative genomic analysis helps to explain genetic evolution, assists in mining for potentially functional gene regions, and has become an important bioinformatics technology in the study of complex gene structures and potential regulatory regions. Using these methods, it was determined that the gene for human carboxylesterase 1 (hCES1) is located on chromosome 16 at position 55,802,851–55,833,186 bp, has a length of 30,336 bps (Gene ID: 1066), and encodes 568 amino acids; the house mouse carboxylesterase 1 (mCES1) gene is located on chromosome 8 at position 93,302,369–93,337,209 bp, has a length of 34,841 bps (Gene ID: 12623), and encodes 566 amino acids; the *Sus scrofa* carboxylesterase APLE isoform gene is located on chromosome 6 at position 27,276,898–27,308,831 bp, has a length of 31,934 bps (Gene ID: 397478), and encodes 565 amino acids. Comparative analysis of the sequences of carboxylesterase in different species using BLAST revealed that the amino acid sequences of porcine PLE-1, PLE-G2, PLE-B9, and PLE-C4 have 77–78% homology with hCES1, 72% with mCES1, and 97% with *Sus scrofa* APLE. The N-terminal signal peptide sequence and C-terminal ER retention signal sequences of the PLE genes are involved in mediation of the proper transport and localization of carboxylesterases. Alignment of the human, mouse, and porcine carboxylesterase amino acid sequences revealed that the signal peptide amino acid sequences differed greatly among different species. C-terminal ER retention signals were also different; the third amino acid from bottomin the ER retention signals in human, mouse, *Sus scrofa* carboxylesterase APLE, and PLE are I, V, A, and A, respectively.

The sequences upstream (-5000 to -1 bp) of the four PLE genes exhibited 99% homology. Using Proscan software(http://www-bimas.cit.nih.gov/molbio/proscan/) to analyse transcription factor binding sites in these regions, relatively few were found in the sequence at -5000 to -2000 bp, whereas several different transcription factor binding sites were found in the region from -2003bp to +1077 bp (see Fig 4 for details). In addition, each type of transcription factor repeatedly appeared at different sites. Details of specific transcription factor binding sites are provided in Table 4.

**Table 4:** The potential transcription factors of -5000bp/-1bp sequence of PLE gene (numbers).

location	potential transcription factor (numbers)
-2003bp/+1077bp	Sp1(18),T-Ag(4),JCV-repeated-sequence(2), GCF(2),TATA (2), EARLY-SEQ1(2),APRT-mouse-US(4),APRT-CHO-US(3), PuF(2), AP-2(7),CTF/NF-1(1), NF-kB(1), ICSbf(1), UCE.2(1).

## 3 Discussion

The complete sequences of four PLE genes, PLE1, PLE-B9, PLE-C4, and PLE-G2, were obtained from a Rongchang pig BAC library. The identification of four isoforms indicates that, if PLE exists in a trimeric form, at least 20 types of PLE trimer could be found in one Rongchang pig. As the library coverage did not reach 100% when screening for PLE-positive clones, it is possible that this study did not identify all PLE-positive clones, and that other PLE genes may also exist.

**Fig 7:**
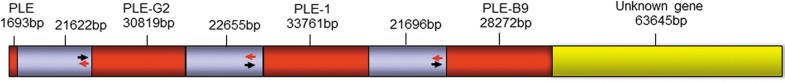
The supposed arrangement of PLE genes at chromosome 6. Note: Red represents PLE genes, Gray represents the region between PLE genes, Yellow represents unknown sequence. The double arrows (red and black) indicate the reverse complementary sequence of PLE regulation regions.

Sequence alignment of the five PLE BAC plasmids (Fig 4) revealed that the same gene sequence was inserted in BAC-206 and BAC-10, differing only slightly at the start and end. The upstream region of BAC-70 and the corresponding region of PLE-G2 in BAC-206 exhibited 99% homology (only 21 gap), indicating that the sequence in BAC-70 is the downstream region of BAC-206, and the downstream region of BAC-206 and the upstream region of BAC-70 overlap. In addition, the gene sequences of PLE-B9 in BAC-70 and BAC-75 exhibited 99% homology, as do their -6082bp to -1bp regions (with only 9 gaps), suggesting that the downstream region of BAC-70 and the upstream region of BAC-75 overlap. In summary, we deduce that the order of the PLE genes on chromosome 6 is PLE-G2, PLE-1, and PLE-B9, which form a PLE gene cluster (Fig 7). PLE-C4, obtained from BAC-119, is located in a different PLE gene cluster. The distance between the two gene clusters wait determination.

The results of the above analyses indicate that the different PLE genes in pigs evolved from the same ancestral gene. In addition, the PLE genes arranged in tandem in chromosomal by multiple gene clusters. The spaces between genes are roughly 22kb in length, and there are very long unknown sequences at the end of the gene cluster (the longest unknown sequence found in this study was 82,549 bps). No simple tandem repeats of the same gene were identified. Furthermore, our results confirm that, in the pig liver carboxylesterase gene family, one gene encodes one polypeptide, which is obviously different compared to antibody gene family.

## 4 Conclusion

In summary, our analyses reveal the complete sequences of four PLE genes, thereby filling the gaps in the database regarding PLE gene sequences and providing the necessary foundation for analysis of the genomic structure, and regulation mechanisms of gene expression of the PLE gene family.

## 5 Materials and Methods

The study was performed in strict accordance with the Guide for the Care and Use of Laboratory Animals Monitoring Committee of Hubei Province, China, and the protocol was approved by the Committee on the Ethics of Animal Experiments at the College of Veterinary Medicine, Huazhong Agricultural University.

### 5.1 Reagents and solutions

#### 5.1.1 Pig genomic BAC library

**Fig 8:**
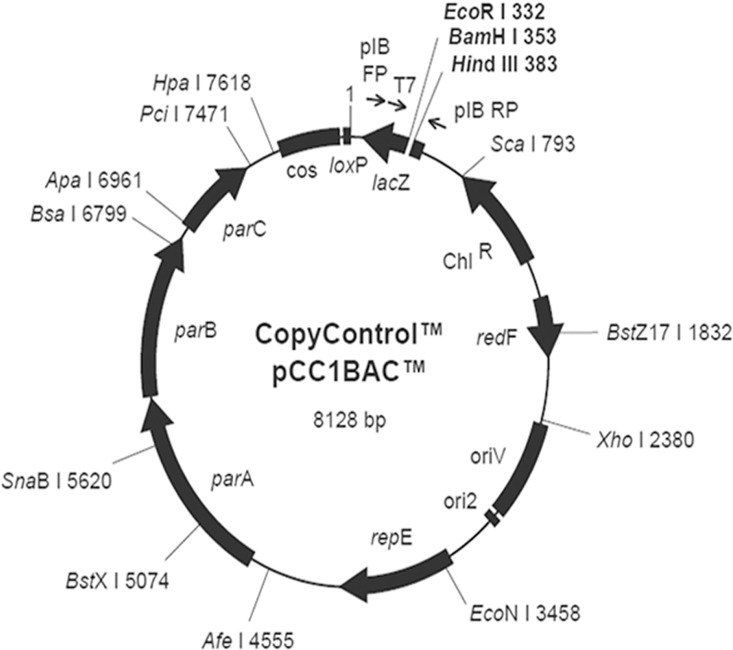
The gene sequence map of vector pCC1BAC.

A Rongchang pig BAC genomic library was selected for use in this study. This library contains a total of 192,000 clones and was constructed from genomic DNA extracted from venous blood of a single Rongchang boar. TransforMax EPI300 *E*.*coli* strain and the pCC1BAC vector (8128 bps) were used for library construction (Fig 8). Inserted gene fragments averaged 116 kb in size, with the majority ranging from 90 to 140 kb. The non-insert rate was 1.4%, and the library covered the porcine genome seven-fold. Analysis of the restriction map generated by *Hind*III digestion of the clones confirmed that the BAC clone s in this library were stable, exhibited low redundancy, and could be used for genetic screening. Our aim was to identify positive clones containing the PLE gene in this BAC library.

#### 5.1.2 Reagents

LA Taq DNA polymerase, dNTPs (TaKaRa, Japan); 5× loading buffer, DL2000, DL1500 (Dongsheng Biotech, China); agarose (BIOWEST, Hong Kong); DNA gel extraction kit (Tiangen Biotech, China); yeast extract, tryptone, NaCl, agar (Oxoid, UK); chloramphenicol (Sigma, USA); BAC/PAC DNA Isolation Kit (OMEGA, USA).

#### 5.1.3 Solutions

Chloramphenicol stock solution: 0.25 g chloramphenicol was dissolved in methanol (99.9% purity) to obtain a 25 mg/ml solution, which was sterilized by filtering through a 0.22 μm filter, aliquoted, and stored at -20°C.

Lysogeny broth (LB) liquid medium: 5 g yeast extract, 10 g tryptone, and 10 g NaCl were dissolved in 800 mL ddH_2_O, and brought to a total volume of 1000 mL. The broth was autoclaved at 121°C for 15 min before use.

LB solid medium: 5 g yeast extract, 10 g tryptone, 10 g NaCl, and 15 g agar were dissolved in 800 mL ddH_2_O and brought to a total volume of 1000 mL. The medium was autoclaved at 121°C for 15 min before use. After cooling the medium to approximately 55°C, chloramphenicol was added on a clean bench (final concentration, 12.5μg/mL). After mixing, the medium was poured into sterile disposable culture plates. The plates were stored at 4°C after medium solidification.

50× TAE nucleic acid electrophoresis buffer: 242 g Tris base and 37.2 g Na-EDTA·2H_2_O were fully dissolved in 700 mL deionized water. Glacial acetic acid (57.1 mL) was added and stirred thoroughly to ensure mixing, and then the total volume was brought to 1 L. The buffer was stored at room temperature before use.

### 5.2 Experimental methods

#### 5.2.1 Primer design

The known γ-PLE cDNA sequence (GenBank: X63323) was analysed along with incomplete PLE gene sequences available in NCBI databases. Based on the AG-GT rule (which states that the first and last two nucleotides of eukaryotic introns are GT and AG, respectively), the PLE coding region was found to be composed of 14 exons. Alignment of partial PLE gene exon and intron sequences using BLAST, demonstrated that some exons of the PLE gene were highly conserved. In addition, the introns of different PLE isoforms showed significant differences in size. Based on these results, eight primers spanning the upstream and downstream junctions of the conserved exons were designed (Table 5).

**Table 5:** The primers for screening PLE positive clones from Rongchang pig BAC library.

ID	Primer(5’-3’)	annealing temperature(°C)	Products length(bps)
**1**	F-CAGGGCAGCCAGCCTCGCCGCCTGTTGTG R-ATGGGAGGGTAGGAGGTGGTGTTCTTCAC	62	208
**2**	F-GCGATGCCCCAGAAGAGGAGGTCAGT R-CAGCTCAGCATGCTTTATCTTGGGTG	62	534
**3**	F-GAACCCCAATGGGGAGGGGCTGCCCC R-CAGCTCAGCATGCTTTATCTTGGGTG	62	181
**4**	F-TACCGCCTGGGCATCTGGGGATTCTTCAG R-AGTTGCCCCGGCTGTGTTCATCCCCTGTG	62	2327/3811
**5**	F-CAGCAGGAGGGGAAAGTGTCTCTGTTCTG R-AAGAGGTTCTTGGCCAAGGGAGACAACAC	62	1540/3558
**6**	F-TCAGGAAGGACATGAAGGCTGCAGCTAAG R-GTTTTACACCCAGCAAGGACAGCAATTTG	62	1869/1213
**7**	F-ACGAGCTCTTGGACTTAACGCTGAAGATG R-TCTCCATGCAAATCAAGAGTTAAAGGTTT	62	2287/4056
**8**	F-ATCTGTGACGGTGGCCCGTCAACACAGAG R-CTCATACATGTAGGTGGGGGCTCCTGCAT	62	3919/4758

#### 5.2.2 Primer verification

PCR amplification was performed using the primers described above with Tongcheng pig genomic DNA (a gift from Professor Liu Bang, Huazhong Agricultural University) as a template. Appropriate primers were identified based on the presence or absence, and size of the resulting amplicons. The PCR system is presented in Table 6. Amplification conditions were as follows: initial denaturation at 94°C for 5 min; followed by 30 cycles of denaturation at 94°C for 30 s, annealing at 62°C for 1 min, and elongation at 72°C for 4 min 50 s, 2.5 min, or 20 s (depending on the primer pair); and a final elongation at 72°C for 10 min. After PCR amplification was completed, 0.8% agarose gel electrophoresis was performed to detect amplicons. If the bands observed by electrophoresis after amplification were similar to the expected sizes of the target products, we concluded that the pair of primers used for amplification was appropriate. Primers that did not amplify products of the expected sizes, or resulted in faint bands were regarded as unsuitable and not used for further analyses.

**Table 6:** Reaction system of PCR.

Ingredients	Sample volume (μL)
pig genome DNA templates (10ng/μL)	0.5
upstream primer (10μM)	1.0
downstream primer(10μM)	1.0
LA Taq enzyme	1.0
dNTP-MIX(10mM)	8.0
10*Buffer(Mg^2+^)	5.0
ddH_2_O	33.5
Total	50.0

#### 5.2.3 Screening of PLE genes

A commonly used three-step PCR screening method, with less stringent conditions, was selected. Primers verified as suitable were used to screen for PLE-positive BAC clones from the Rongchang pig BAC genomic DNA library. The detailed procedure was as follows:

1. The library (consisting of 500 384-well plates) was removed from storage at -80°C and placed at 4°C to thaw. A 384-pin replicator was used to inoculate LB agar plates containing 12.5μg/mL chloramphenicol with all BAC clones from the 384-well plates. LB plates were incubated overnight at 37°C and colony growth observed. Next, 5 mL of sterile LB liquid medium containing 12.5μg/mL chloramphenicol was added to each LB agar plate. After evenly spreading and mixing the clones with the medium, using a sterile triangular cell spreader, the broth mixture was tipped to one side of the culture plate, aspirated with a sterile pipette tip, and transferred to an EP tube for later use.
2. Each 384 culture plate colony mixture from the previous step was transferred to a LB liquid medium (containing 12.5μg/mL chloramphenicol). The culture was expanded by overnight culture at 37°C with shaking, and the BAC DNA harvested using the BAC/PAC DNA Isolation Kit (OMEGA, USA).
3. PCR amplification was performed using the BAC DNA clone mixture from the 384-well plates as templates (for reaction conditions see section 5.2.2). PCR amplification products were characterised by 0.8% agarose gel electrophoresis and those 384-well plates containing positive clones were subjected to further screening.
4. 384-well plates containing positive clones were collected, and a 384-pin replicator was used to inoculate all BAC clones onto LB agar plates containing 12.5μg/mL chloramphenicol, followed by incubation overnight at 37°C with shaking. Colony growth was observed, and care was taken to ensure colonies were not so large as to merge together and cause cross-contamination.
5. Sterile water (400 μL) was added to 24 1.5 mL centrifuge tubes. A toothpick was used to transfer each set colonies (16 colonies in a set) to the centrifuge tubes, and these samples were then boiled for 5 min. PCR amplification was performed directly using this mixture as the template (for reaction conditions see section 5.2.2). One toothpick was used to transfer a sample from each single colony, while taking care to avoid cross-contamination with other colonies from the LB agar medium. The LB culture plates with the remaining colonies were stored at 4°C. PCR amplicons were characterised by 0.8% agarose gel electrophoresis, to identify colonies positive for PLE.
6. Sixteen colonies containing positive clones were identified. The remaining colonies that had been stored on LB agar medium (see **e** above) were used as templates for colony PCR (for reaction conditions see section 5.2.2) and amplicons were characterised by 0.8% agarose gel electrophoresis, to confirm wells containing single positive clones.
7. Colonies identified as containing positive clones were transferred to LB liquid medium containing 12.5μg/mL chloramphenicol and incubated at 37°C with shaking (200 rpm) overnight (16 h) to expand the culture, and obtain cultures of BAC clones containing PLE genes.

#### 5.2.4 Identification and sequence analysis of positive BAC clones

BAC DNA clones were isolated using the BAC/PAC DNA Isolation Kit (OMEGA, USA). To measure DNA concentration and purity, the plasmids were sent to Tianjin Biochip Corporation (Tianjin, China) and Biomarker Technologies (Beijing, China) after characterisation by 0.8% agarose gel electrophoresis, where they were further purified and concentrated to 69ng/μL with a total DNA of 34.5 μg. Using PacBio third-generation sequencing, each BAC was allocated to a single sequencing unit; a total of five sequencing units were used, in order to ensure accurate separation of sequencing data.

PacBio third-generation sequencing produces an average read length of 10–15 kb, and a single sequencing unit can process up to 1 Gb [16]. In 2015, researchers at the Donald Danforth Plant Science Center employed the PacBio RS II sequencing system to analyse the 245 Mb genome of *Oropetium thomaeum*with 72-fold coverage. A draft genome was constructed which contained telomeric and centromeric sequences, long terminal repeats, retrotransposons, tandem repeat genes, and other genomic elements that are difficult to sequence, with over 99.999% accuracy [17]. These data demonstrate that this sequencing method can produce high-quality, complete, and reliable genomic data.

## References

[1] Morris AP, Brain KR, Heard CM. Synthesis of haloperidol prodrugs and their hydrolysis by porcine liver esterase. Drug Metab Lett. 2008, 2(4): 275–279. 10.2174/187231208786734111 . 19356105

[2] Roberti M, Rondanin R, Ferroni R, et al Pig liver esterase (PLE) mediated resolution of N-substituted 4-benzoyloxy-3-carbomethoxypiperidines: a convenient preparation of 4-hydroxy-and 4-benzoyloxy-3- carbometho- xypiperidines in enantiomerically pure form. Tetrahedron Asymmetry, 2000, 11, 4397–4405. 10.1016/S0957-4166(00)00404-3

[3] Brüsehaber E, Böttcher D, Bornscheuer UT. Insight into the physiological role of pig liver esterase: Isoenzymes show differences in the demethylation of prenylated proteins. Bioorg Med Chem. 2009, 17(23): 7878–7883. 10.1016/j.bmc.2009.10.033 . 19884014

[4] Liu ZQ, Liu WF, Huang YP, et al Lipopolysaccharide significantly influences the hepatic triglyceride metabolism in growing pigs. Lipids in Health and Disease, 2015, 14 (64):1–10. 10.1186/s12944-015-0064-8 . 26121977 PMC4495945

[5] Junge W, Heymann E. Characterization of the Isoenzymes of Pig-Liver Esterase. 2. Kinetic Studies. Eur J Biochem, 1979, 95(3): 519–525. . 446478 10.1111/j.1432-1033.1979.tb12992.x

[6] Adler AJ, Kistiakowsky GB. Isolation and properties of pig liver esterase. J BiolChem, 1961, 236(12): 3240–3245. . 13859431

[7] Farb D, Jencks WP. Different forms of pig liver esterase. Arch BiochemBiophys, 1980, 203(1): 214–226. 10.1016/0003-9861(80)90171-x . 7406498

[8] Hummel A, Brüsehaber E, Böttcher D, et al Isoenzymes of pig-liver esterase reveal striking differences in enantioselectivities. Angew Chem Int Ed EngI. 2007; 46(44): 8492–8494. 10.1002/anie.200703256 . 17902087

[9] Lange S, Musidlowska A, Schmidt-Dannert C, et al Cloning, functional expression, and characterization of recombinant pig liver esterase. Chembiochem. 2001, 2(7–8): 576–582. 10.1002/1439-7633(20010803)2:7/8<576::aid-cbic576>3.0.co;2-y . 11828491

[10] Hermann M, Kietzmann MU, Ivancic M, et al Alternative pig liver esterase (APLE)-cloning, identification and functional expression in Pichia pastoris of a versatile new biocatalyst. J Biotechnol 2008, 133(3): 301–310. 10.1016/j.jbiotec.2007.10.010 . 18078679

[11] Musidlowska-Persson A, Bornscheuer UT. Recombinant porcine intestinal carboxylesterase: Cloning from the pig liver esterase gene by site-directed mutagenesis, functional expression and characterization. Protein Eng. 2003; 16(12): 1139–1145. 10.1093/protein/gzg120 . 14983097

[12] David L, Guo XJ, Villard C, et al Purification and molecular cloning of porcine intestinal glycerol-ester hydrolase—evidence for its identity with carboxylesterase. Eur J Biochem. 1998; 257(1):142–148. 10.1046/j.1432-1327.1998.2570142.x . 9799112

[13] Musidlowska A, Lange S, Bornscheuer UT. By overexpression in the yeast Pichia pastoris to enhanced enantioselectivity: new aspects in the application of pig liver esterase. Angew Chem Int Ed EngI. 2001; 40(15): 2851–2853. 10.1002/1521-3773(20010803)40:15<2851::aid-anie2851>3.0.co;2-v . 29712000

[14] Hasenpusch D, BornscheuerU T, Langel W. Simulation on the structure of pig liver esterase. J Mol Model. 2011, 17(6): 1493–1506. 10.1007/s00894-010-0846-x . 20862595

[15] Liu L, Yin J, Li W, et al Construction of a bacterial artificial chromosome library for the Rongchang pig breed and its use for the identification of genes involved in intramuscular fat deposition. Biochemical and biophysical research communications, 2010, 391(2): 1280–1284. 10.1016/j.bbrc.2009.12.060 . 20018173

[16] Eid J, Fehr A, Gray J, et al Real-time DNA sequencing from single polymerase molecules. Science, 2009, 323(5910): 133–138. 10.1126/science.1162986 . 19023044

[17] VanBuren R, Bryant D, Edger PP, et al Single-molecule sequencing of the desiccation-tolerant grass Oropetium thomaeum. Nature. 2015; 527(7579):508–11. 10.1038/nature15714 . 26560029

